# Sleep architecture, obstructive sleep apnea and functional outcomes in adults with a history of Tick-borne encephalitis

**DOI:** 10.1371/journal.pone.0246767

**Published:** 2021-02-08

**Authors:** Malin Veje, Marie Studahl, Erik Thunström, Erika Stentoft, Peter Nolskog, Yeliz Celik, Yüksel Peker

**Affiliations:** 1 Department of Infectious Diseases, Institute of Biomedicine, Sahlgrenska Academy, University of Gothenburg, Gothenburg, Sweden; 2 Institute of Specific Prophylaxis and Tropical Medicine, Medical University Vienna, Vienna, Austria; 3 Department of Infectious Diseases, Region Västra Götaland, Sahlgrenska University Hospital, Gothenburg, Sweden; 4 Department of Molecular and Clinical Medicine/Cardiology, Sahlgrenska Academy, University of Gothenburg, Gothenburg, Sweden; 5 Department of Communicable Disease Control and Prevention, Region Västra Götaland, Gothenburg, Sweden; 6 Koc University Research Centre for Translational Medicine (KUTTAM), Istanbul, Turkey; 7 Department of Clinical Sciences, Respiratory Medicine and Allergology, Faculty of Medicine, Lund University, Lund, Sweden; 8 Division of Pulmonary, Allergy, and Critical Care Medicine, University of Pittsburgh School of Medicine, Pittsburgh, PA, United States of America; Brigham and Women’s Hospital and Harvard Medical School, UNITED STATES

## Abstract

Tick-borne encephalitis (TBE) is a widespread viral infection of the central nervous system with increasing incidence in Europe and northern Asia. Post-infectious sequelae are frequent, and patients with TBE commonly experience long-term fatigue and subjective sleep disturbances. Obstructive sleep apnea (OSA) may be a contributing factor, and objective sleep studies with polysomnography (PSG) are lacking. Forty-two adults, 22 TBE patients (cases), diagnosed in Region Västra Götaland, Sweden, between 2012 and 2015, and 20 controls without a known TBE history, underwent an overnight PSG, respectively. All participants responded to questionnaires. The cases and controls were similar regarding age, sex, obesity, concomitant diseases, smoking, and alcohol habits. Despite similar PSG characteristics such as total sleep time and OSA severity indices, the TBE cases reported statistically more sleep-related functional impairment on the Functional Outcome of Sleep Questionnaire (FOSQ) compared with the controls (median scores 18.1 *vs*. 19.9; *p*<0.05). In a multivariate analysis, TBE correlated significantly with the lower FOSQ scores (unstandardized β −1.80 [%95 confidence interval −3.02 - −0.58]; *p = 0*.*005*) independent of age, sex, total sleep time and apnea-hypopnea-index. TBE cases with OSA reported the lowest scores on the FOSQ compared with the other subgroups with TBE or OSA alone, and the ones with neither TBE nor OSA. TBE is associated with impaired functional outcomes, in which concomitant OSA may worsen the subjective symptoms. Further studies are warranted to determine the effect of treatment of concomitant OSA on functional outcomes with regard to optimal rehabilitation of TBE.

## Introduction

Tick-Borne Encephalitis (TBE), caused by Tick-Borne Encephalitis virus (TBEV), is one of the most frequent viral central nervous system (CNS) infections in Europe and Asia, with an increasing incidence in many areas, including Sweden [1]. Post mortem studies of TBE infected brains display inflammation in the spinal cord, brainstem, basal ganglia, thalamus, and cerebellum [2]. Although imaging findings are only occurring in about a fifth of patients, the most common MRI lesions are localized in subcortical regions such as the thalamus and basal ganglia [3, 4], parts of the brain important for the regulation of”general arousal” and attention [5]. The severity of TBEV infections varies greatly, from asymptomatic infections to severe encephalomyelitis. The mortality is low. Nevertheless, neurological and/or neuropsychological long-term sequelae are common [6]. In a previous study, we found that TBE patients suffered from significantly more tiredness/fatigue than a matched control group, and that the sleep-related quality of life measured by self-assessment with the Functional Outcome of Sleep Questionnaire (FOSQ) affected their social life [7]. Several other studies have presented self-reported sleep disturbances [6, 8–10], and post infectious fatigue [6, 8, 11, 12] after TBE, but the cause of these symptoms has not been sufficiently clarified. Obstructive sleep apnea (OSA), which is a common cause of excessive daytime sleepiness and impaired sleep-related functional outcomes [13] has yet not been investigated among adults with a history of TBE. In OSA patients, alterations in several areas in the brain, such as the Optical Frontal Cortex (OFC), hippocampus, and cerebellum have been detected, but the question remains whether these defects are caused by the OSA or vice versa [14].

The aim of the present study was to study sleep architecture and occurrence of OSA on polysomnography (PSG) among patients previously diagnosed with TBE, compared with a control group, and investigate the association between TBE and sleep related functional outcomes, adjusted for the polysomnographic variables and concomitant OSA.

## Materials and methods

The study complied with the Declaration of Helsinki and was approved by the Gothenburg Ethics Committee (application number 884–14).

### Study participants

**Fig 1:**
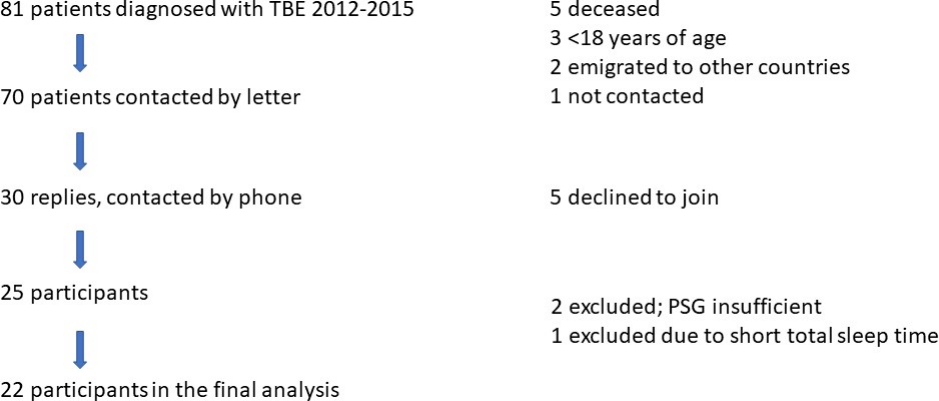
Flow chart of the included and excluded TBE patients in the study. *Definition of abbreviations*: PSG = Polysomnography; TBE = Tick-Borne Encephalitis.

Data on TBE cases from Region Västra Götaland during the period January 2012- October 2015 was retrieved from the Regional center for prevention of communicable diseases. As shown in Fig 1, 81 patients were diagnosed with TBE during that time period, of which 70 were contacted by letter (five were deceased, three were <18 years of age, two had emigrated to other countries, and one was mistakenly not contacted). In all, 30 patients responded, and were contacted by telephone by M.V. After oral information, five people declined to join the study, leaving 25 TBE patients for participation after oral and written consent.

For the control group, 20 adults without a history of TBEV infection were included after oral information and written consent. Twelve persons were retrieved from ongoing population studies [15], and eight individuals from the staff of the Departments of Infectious Diseases and Internal Medicine, Sahlgrenska University Hospital/Östra, Gothenburg.

### Polysomnography

Polysomnography (PSG) is the gold standard to diagnose obstructive sleep apnea (OSA) [16]. In our study, a full-night PSG recording was performed using the NOX-A1 system (Nox Medical Inc., Reykjavik, Iceland). The PSG recording included an electroencephalogram (F4/M1, F3/M2, C4/M1, C3/M2, O2/M1, O1/M2), electrooculogram, submental and tibialis electromyograms, as well as electrocardiogram. Ventilatory monitoring included a nasal pressure detector using a nasal cannula/pressure transducer system, and thoracoabdominal movement detection through two respiratory inductance plethysmography belts. A finger pulse-oximeter detecting heart rate and oxyhemoglobin saturation (SpO2) as well as body position and movement detection were also included. Moreover, snoring was recorded by a built-in microphone in the NOX-A1 device, which provided the opportunity to play the audio along with the other recorded signals during manual scoring of the events. A specifically trained nurse attached all the electrodes and monitoring equipment required at the Department of Internal Medicine, Sahlgrenska University Hospital/ Östra, Gothenburg between 15.00–16.00 h, instructed the patient to start the recordings at bedtime/ 22.00h, and unattended home-based PSG took place at the same department in all cases, and in eleven out of 20 controls. Due to logistic and practical reasons, the remaining nine PSGs were performed in the participants’ home environment. The PSG recordings of the cases were performed from May 2016 to March 2017 (after a median 2.0 years, interquartile range [IQR], 2.0–5.0 years, following the TBEV infection), and the PSGs of the control group were conducted between November 2018 and March 2020. Participants with a total sleep time of less than 4 h were offered a new PSG. Sleep stages and arousals were scored based on 30-second epochs in accordance with The American Academy of Sleep Medicine (AASM) Manual for the Scoring of Sleep and Associated Events 2.5 [17] Apnea was defined as an almost complete (≥ 90%) cessation of airflow, and hypopnea was defined as a decrease in nasal pressure amplitude of ≥ 30% and/or thoracoabdominal movement ≥ 30% for ≥ 10 s if there was a significant oxyhemoglobin desaturation (reduction by ≥3% from the immediately preceding baseline value), and/or an arousal, according to the latest recommendations of the AASM [18]. Furthermore, the total number of significant desaturations was scored, and the oxygen desaturation index (ODI) was calculated as the number of significant desaturations per hour of total sleep time. Minimum SpO2 and time spent below 90% SpO2 (TS90%) values were also recorded. OSA was defined as an apnea-hypopnea index (AHI) ≥ 15 events/h of the total sleep time, based on the latest International Classification of Sleep Disorders– 3 [19], when OSA-related symptoms are absent. The subjects with AHI 5–15 events/h with symptoms were categorized as no-OSA in order to be consequent with the AHI-based classification. All PSG recordings were coded and manually scored in a mixed order by a certified sleep technician blinded to the group allocation (case/control), demographics, clinical characteristics, and results of the questionnaires during a 4-week period in May-June 2020.

### Questionnaires

All patients and controls were asked to answer the following questionnaires: Epworth Sleepiness Scale (ESS), Likert scales (fatigue and dyspnea), Functional Outcome of Sleep Questionnaire (FOSQ), Alcohol Use Disorders Identification Test (AUDIT), and a questionnaire according to the clinical routines about concomitant diseases, smoking and sleeping habits.

FOSQ is a validated [20] questionnaire regarding sleep-related symptoms, divided into five dimensions: activity level, vigilance, intimacy and sexual relationships, general productivity, and social outcome. Item responses range from no difficulty (4 points) to extreme difficulty (1 point). The total score is obtained by the sum of the subscale scores, and the total score ranges from 5 to 20 with lower scores suggesting greater function impairment. The impairment in the FOSQ is defined as a total score less than 17.9 points [21]. The Swedish translation of FOSQ has been validated in research [22].

ESS measures the patient´s self-assessed daytime sleepiness, and records the likelihood of dozing off in eight different daily situations with responses “would *never* doze”, 0 point; “*slight* chance of dozing”, 1 point; “*moderate* chance of dozing”, 2 points; or “*high* chance of dosing”, 3 points; resulting in a total score of 0–24 points [23]. Excessive Daytime Sleepiness was defined as an ESS score of at least 11 points [23]. The Swedish translation of the ESS was approved by the Swedish Sleep Research Society in 2001 [24], and has been widely used in clinical routines and research in Sweden, albeit not validated.

Different types of five-graded Likert scales for self-assessment of symptoms are widely used [25]. The scales for the investigation of fatigue and dyspnea yield a total score of 1 (“not at all”) -5 (“very much”) points. Scores of 4 or 5 are defined as being suggestive for fatigue and dyspnea, respectively.

The AUDIT questionnaire, developed by the World Health Organization (WHO), consists of ten questions regarding alcohol drinking habits designed to identify risk-drinking, and is broadly used all over the world. The ten items are multiplied with a frequency factor of 0–4, resulting in a total score of 0–40 points, where 8 points for men and 6 points for women indicate a risk behavior [26, 27]. The Swedish translation of the AUDIT was done by Professor Hans Bergman at Karolinska Institute, Sweden [28], and has been broadly used in clinical routines and research in Sweden, though not validated.

### Statistics

For descriptive statistics, variables were reported as mean and standard deviation, or median and IQR, 25^th^ and 75^th^ percentile for continuous variables, and as counts and percent for categorical variables. The baseline differences between groups were tested by Independent-Sample T-Test or Mann Whitney U when appropriate for the continuous data, and by the Chi-square test for the categorical data. Shapiro Wilk test was used to test normality assumption of the current data for all variables. Kruskal Wallis test was conducted to compare the study subgroups based on the combination of presence or absence of TBE and OSA. Mann Whitney U test with Bonferroni correction was applied for post-hoc pairwise comparisons.

A multivariate linear regression was performed to test variables associated with the primary outcome, which was the total FOSQ score. The covariates were selected as recommended in a recent guidance [29]. TBE diagnosis, sex, age, PSG variables (total sleep time, percentage of Rapid Eye Movement (REM) and Slow-Wave sleep (sleep stage N3) as well as Arousal Index and Apnea Hypopnea Index (AHI)) were included as covariates in the model.

All statistical tests were two-sided, and a p-value < .05 was considered significant. Statistical analysis was performed using SPSS^®^ 26.0 for Windows^®^ (SPSS Inc., Chicago, Illinois, USA).

## Results and discussion

### Study participants

Twenty-five patients were initially included in the study. Due to technical problems with the pulse oximeter, the first three recordings had to be withdrawn from the study, and only one participant out of three was able to return for a second recording. One patient with a total sleep time of 85 minutes did not want to re-do the sleep recording, and was therefore excluded. Thus, PSG recordings of 22 TBE patients were included in the study (Fig 1). The demographic characteristics of the patients compared with the 20 control subjects are presented in Table 1. There were no major differences between the two study groups regarding background factors such as age, sex, obesity, smoking and alcohol habits. Few participants had concomitant diseases; hypertension, atrial fibrillation, lung disease, diabetes, and anti-depressive medication were more common in the control group, whereas substituted hypothyroidism (4/22 patients) was more frequent among the TBE patients. One patient was treated with disulfiram (Antabuse) for history of alcohol dependency. None of the TBE cases had a history of cataplexy.

**Table 1:** Demographic and clinical characteristics of the study population.

	TBE Cases n = 22	Controls n = 20
Age, years	53.7 (15.3)	50.4 (15.5)
Male sex, %	68.2	55.0
BMI, kg/m^2^	26.2 (4.0)	25.5 (4.1)
Obesity, %	13.6	10.0
Current smoking, %	9.1	5.0
Hypertension, %	18.2	30.0
CAD, %	9.1	5.0
Atrial fibrillation, %	0.0	10.0
Lung disease, %	4.5	15.0
Diabetes, %	0.0	5.0
Hypothyroidism, %	18.2	0.0
Anti-depressive medication, %	4.5	5.0

*Definition of the abbreviations*: BMI = Body Mass Index; CAD = Coronary Artery Disease; TBE = Tick-Borne Encephalitis.

Continuous data are presented as mean with standard deviation.

Categorical data are presented as percentage.

### Polysomnography results

The comparison of the PSG parameters between patients and controls is shown in Table 2. The total sleep time (TST) was almost identical in the two groups, with a mean sleep time of 385 and 388 minutes, respectively. There were no statistically significant differences between the TST values of the participants undergoing unattended PSG in-hospital (n = 33) *vs* at home (n = 9). Other PSG parameters including the proportion of the sleep stages as well as OSA severity indices (AHI and ODI) also did not differ between the TBE patients and controls, whereas percent time spent below 90% SpO_2_ as well as min SpO_2_ were significantly lower in the TBE group (Table 2). When applying an AHI cut-off value of 15 events/hour, regardless of daytime sleepiness, the occurrence of OSA was slightly higher in the TBE group but the difference was not statistically significant (Table 3). There were five participants with AHI ≥5 and <15 events/h, and ESS ≥11 (2 out of 7 among the TBE cases, and 3 out of 8 among controls; not significant). There were four patients with hypothyroidism, all had an AHI <15 events/hour.

**Table 2:** Polysomnographic characteristics of the study population.

	TBE Cases n = 22	Controls n = 20
Total sleep time, min	385.3 (82.9)	387.9 (49.7)
Sleep latency, min	16.4 (3.7–28.8)	11.3 (6.3–17.1)
REM latency, min	87.4 (51.1)	90.7 (38.1)
WASO, min	60.0 (40.6–95.6)	59.5 (31.7–93.6)
Sleep efficiency, %	83.6 (73.0–89.8)	85.0 (79.4–91.1)
Stage N1, min	38.3 (22.0–81.6)	46.5(34.4–70.1)
Stage N1% of TST	10.3 (6.6–20.0)	12.8 (8.3–19.9)
Stage N2, min	203.5 (44.6)	204.4 (45.2)
Stage N2% of TST	53.7 (10.7)	52.6 (8.5)
Stage N3, min	57.5 (37.4)	62.7 (43.9)
Stage N3% of TST	14.3 (9.1)	16.2 (11.0)
Stage REM, min	75.6 (33.8)	67.1 (20.3)
Stage REM % of TST	19.0 (6.3)	17.3 (5.1)
Total Arousals	125.0 (64.0–192.5)	119.5 (76.0–179.5)
Arousal Index, events/h	18.4 (8.2–35.8)	18.7 (11.0–31.7)
Supine time, min	152.1 (96.7)	150.2 (102.6)
Supine time % of TST	39.5 (24.7)	38.2 (26.6)
AHI, events/h	13.2 (5.5–38.0)	12.9 (6.7–29.3)
Supine AHI, events/h	21.4 (5.4–66.3)	17.3 (2.8–46.8)
Non-supine AHI, events/h	6.0 (2.0–17.1)	4.4 (1.1–18.5)
Rem-AHI, events/h	17.1 (7.1–47.6)	17.4 (7.5–38.8)
Non-REM-AHI, events/h	10.3 (4.7–32.7)	11.0 (3.6–28.1)
ODI, events/h	10.5 (3.1–20.7)	6.5 (3.1–12.7)
Mean SpO_2_%	93.8 (92.7–94.7)	94.4 (93.2–94.8)
Time spent <90% SpO2%*	1.4 (0.1–8.6)	0.2 (0.0–0.6)
Min SpO_2_%*	85.0 (81.0–88.5)	89.0 (86.0–90.0)

*Definition of the abbreviations*: AHI = Apnea Hypopnea Index; ODI = Oxygen Desaturation Index; REM = Rapid Eye Movement; SpO_2_ = Oxyhemoglobin saturation; TBE = Tick-Borne Encephalitis; WASO = Wake After Sleep Onset.

Continuous data are presented as mean and standard deviation, or median with 25^th^ and 75^th^ percentiles (metric variables).

*p<0.05

**Table 3:** Comparison of the outcomes of the study population.

	TBE Cases n = 22	Controls n = 20
Likert scale fatigue	2.0 (1.0–5.0)	1.0 (1.0–2.0)
Fatigue*, %	31.8	5.3
Likert scale dyspnea	2.0 (1.0–2.0)	1.0 (1.0–2.0)
AUDIT score	3.0 (1.8–5.0)	3.0 (2.0–4.0)
FOSQ score*	18.1 (16.4–19.2)	19.9 (19.4–20.0)
Impaired FOSQ (<17.9)*, %	40.9	10.0
ESS score*	9.4 (3.5)	6.8 (4.1)
EDS* (ESS score≥11), %	54.5	20.0
OSA (AHI≥15 events/h), %	45.5	40.0

*Definition of the abbreviations*: AHI = Apnea Hypopnea Index; AUDIT = Alcohol Use Disorders Identification Test; EDS = Excessive Daytime Sleepiness (ESS score ≥10); ESS = Epworth Sleepiness Scale; FOSQ = Functional Outcome of Sleep Questionnaire; OSA = Obstructive Sleep Apnea; TBE = Tick-Borne Encephalitis.

Continuous data are presented as mean with standard deviation, or median with 25^th^ and 75^th^ percentiles.

Categorical data are presented as percentage.

*p<0.05

### Questionnaire results

**Fig 2:**
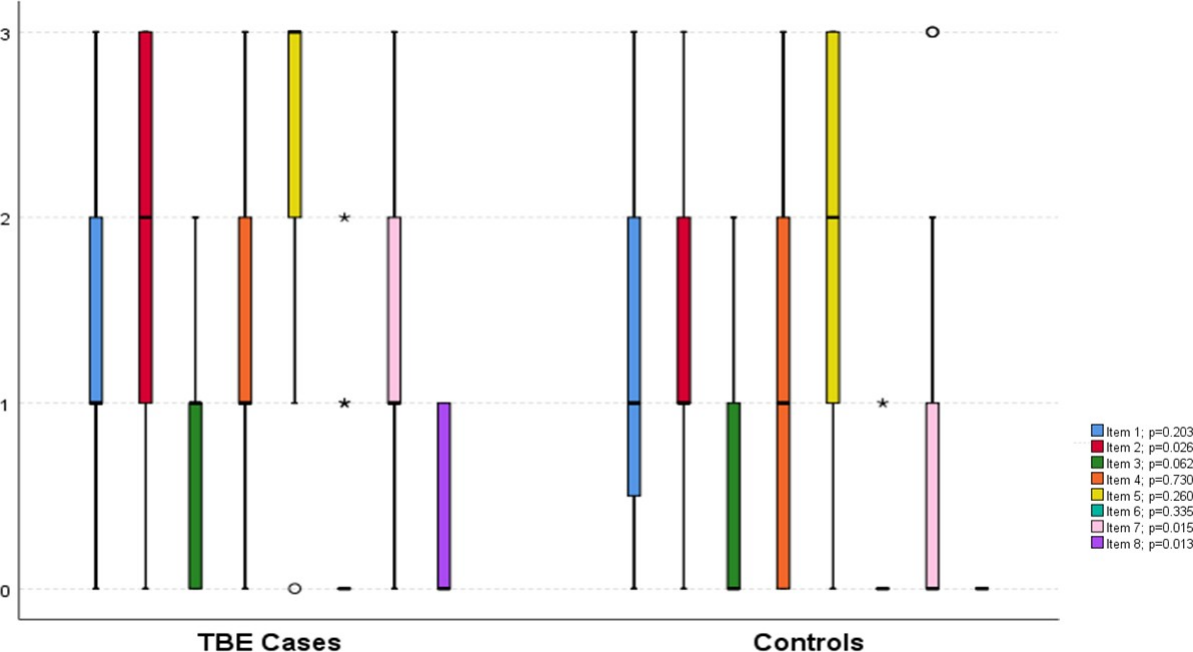
Comparison of the Epworth Sleepiness Scale items among the cases and controls. Box and whiskers plot where the lines show median and interquartile ranges, whiskers show minimum and maximum values, circles depict outliers, stars extreme outliers. The questions refer to chance of dozing off during different situations: *Item 1*, while sitting and reading; *Item 2*, while watching TV; *Item 3*, while sitting inactive in a public place (e.g. theatre or a meeting); *Item 4*, as a passenger in a car for an hour without a break; *Item 5*, while lying down to rest in the afternoon when circumstances permit; *Item 6*, while sitting and talking to someone, *Item 7*, while sitting quietly after a lunch without alcohol; *Item 8*, in a car, while stopped for a few minutes in the traffic. 0 refers to “never”, 1 to “slight chance”, 2 to “moderate chance”, and 3 to “high chance” of dozing off.

**Fig 3:**
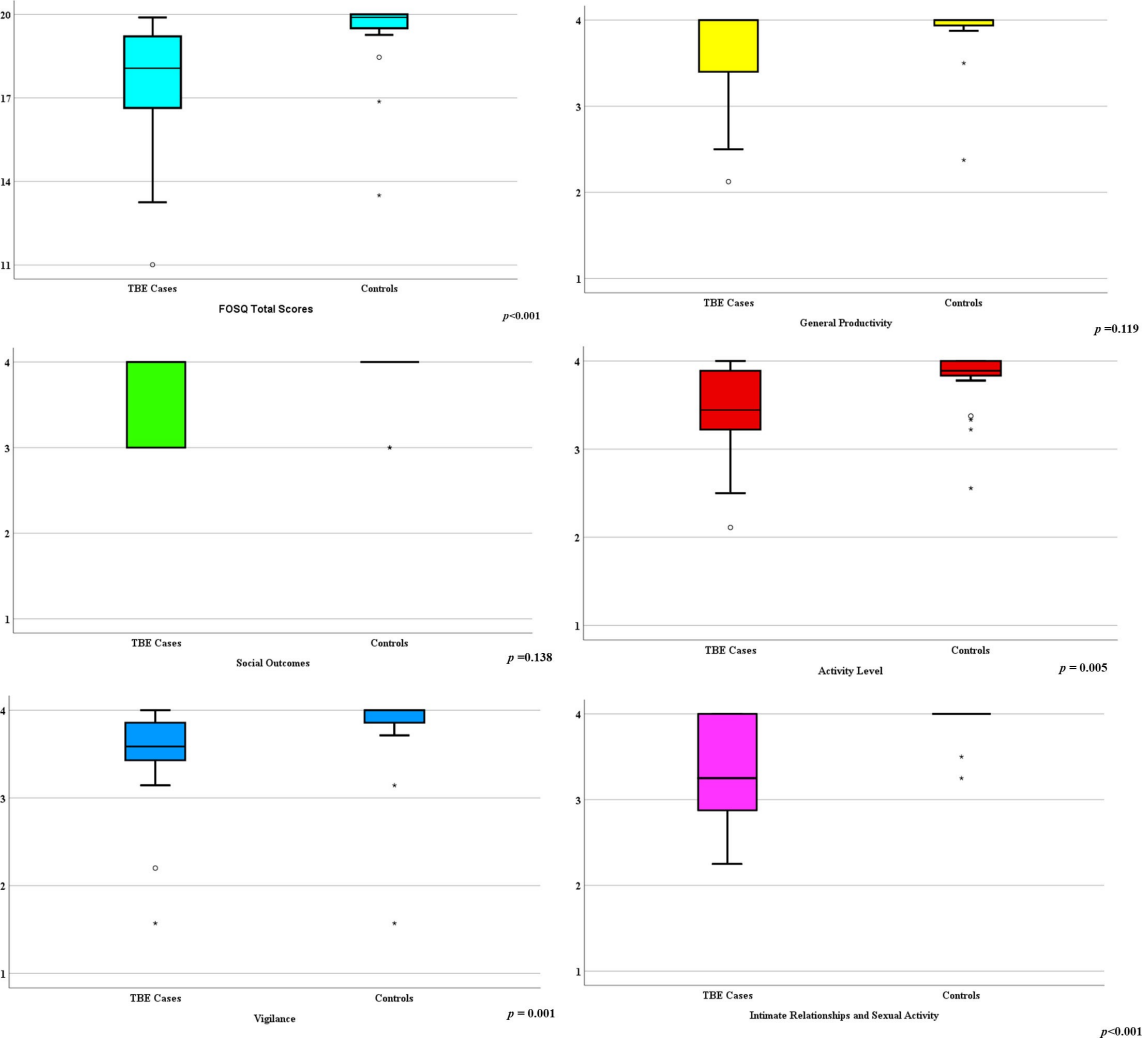
Comparison of the total (3a) and subdimension results (3b-f) of the Functional Outcome of Sleep Questionnaire among the cases and controls. Box and whiskers plot where the lines show median and interquartile ranges, whiskers show minimum and maximum values, circles depict outliers, stars extreme outliers. *Definition of abbreviations*: FOSQ = Functional Outcome of Sleep Questionnaire.

Based on the Likert fatigue scale score ≥4 points, seven of the patients demonstrated fatigue compared with only one of the controls (*p<0*.*05*) (Table 3). There was no statistical difference between the groups on the Likert dyspnea scores. Regarding the ESS scores, the TBE patients demonstrated significantly higher values than did the controls (average 9.4 *vs* 6.8 points); and Excessive Daytime Sleepiness was significantly higher among the TBE patients compared with the controls (55% *vs* 20%, respectively) (Table 3). As illustrated in Fig 2, the median scores differed significantly between the TBE cases and controls on the items 2, 7 and 8, respectively. The TBE patients scored statistically lower in the total FOSQ scores than did the controls, and the subdimensions Activity Level and Vigilance as well as Intimate Relationships and Sexual Activity were significantly lower in the TBE group while General Productivity and Social Outcomes did not differ significantly (Fig 3A–3F).

**Fig 4:**
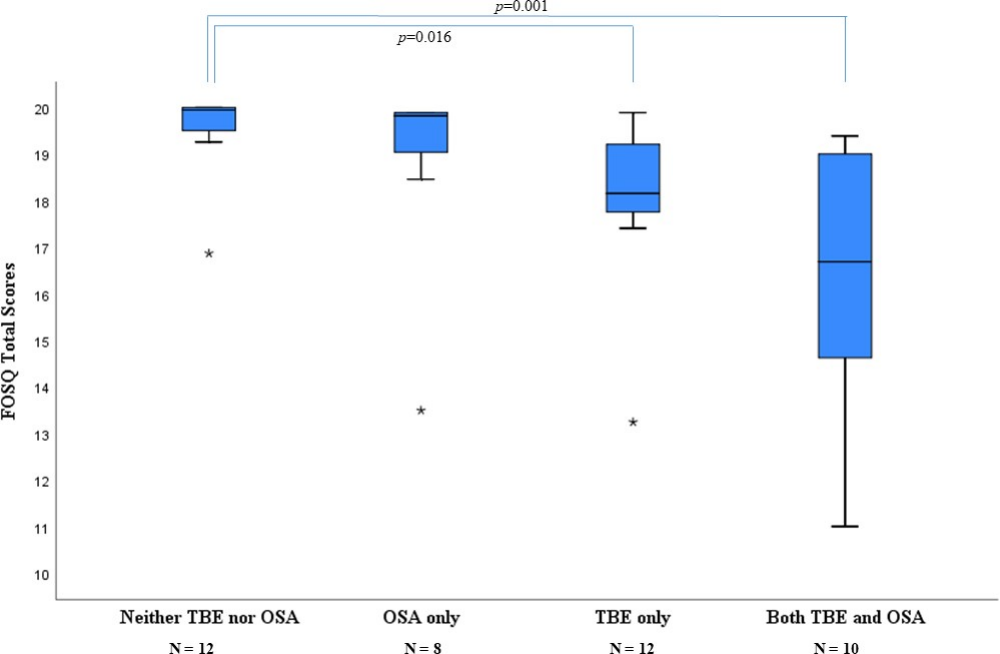
Comparison of the total score in the Functional Outcome of Sleep Questionnaire in four subgroups of the study participants. Box and whiskers plot where the lines show median and interquartile ranges, whiskers show minimum and maximum values, circles depict outliers, stars extreme outliers. *Definition of abbreviations*: FOSQ = Functional Outcome of Sleep Questionnaire; OSA = Obstructive Sleep Apnea; TBE = Tick-Borne Encephalitis.

As shown in Table 4, total sleep time and age were positively correlated with the FOSQ scores, whereas history of TBE and severity of OSA in terms of AHI were inversely correlated in a multivariate linear regression model. As illustrated in Fig 3, the patients with TBE and concomitant OSA demonstrated the lowest FOSQ scores compared with the other subgroups with TBE or OSA alone, and the ones with neither TBE nor OSA. The cases with TBE only also demonstrated significantly lower scores than the ones with neither TBE nor OSA (Fig 4).

**Table 4:** Significant variables associated with the FOSQ total scores in a multivariate linear regression analysis.

	Unstandardized β	95% Confidence Interval for		*p values*
		Lower Bound	Upper Bound	
TBE	- 1.80	- 3.02	- 0.58	0.005
Age, years	0.07	0.01	0.12	0.016
TST, minutes	0.01	0.01	0. 12	0.026
Male sex	- 0.07	-1.50	1.37	0.925
AHI, events/hour	- 0.06	- 0.12	- 0.01	0.029
AI, events/hour	0.01	- 0.04	0.07	0.662
REM sleep, %	- 0.06	- 0.18	0.07	0.367
Stage N3, %	- 0.02	- 0.10	0.05	0.430

*Definition of the abbreviations*: AHI = Apnea Hypopnea Index; AI = Arousal Index; FOSQ = Functional Outcome of Sleep Questionnaire; REM = Rapid-Eye Movement; TBE = Tick-Borne Encephalitis; TST = Total Sleep Time.

To the best of our knowledge, this is the first study addressing the occurrence of sleep-related functional outcomes as well as OSA in TBE patients, based on an objective evaluation with overnight PSG investigations. We found OSA in a large proportion of the study population, both among the cases and the controls. According to a recent review, 34% of men and 17% of women in a general population in the USA are estimated to be affected by OSA (AHI ≥15) [30]. Important risk factors for OSA are obesity and male sex. It is puzzling, that despite a lack of obesity in both groups (mean BMI 26,2 and 25,5, respectively), our study population was to a large extent diagnosed with OSA. One contributing factor could be an inclusion bias, namely, that both TBE patients and controls with a suspicion of OSA were more inclined to volunteer for the study. Although our findings need to be confirmed in larger cohorts, the fact that almost 50% of the TBE patients suffered from OSA, indicates that this treatable condition [30] could be underdiagnosed in the group of patients with post encephalitic fatigue. Co-morbidities among the study participants may have influenced the results; one patient was treated with disulfiram (Antabuse), a medication linked to decreased REM sleep [31], and four patients had substituted hypothyroidism, a disease connected to sleep apnea when not sufficiently treated [32], however, neither of the four hypothyroidism patients in our study had an AHI above 15 events/hour.

Despite a similar proportion of OSA in the TBE and control group, the patients stated significantly more self-assessed daytime sleepiness/ fatigue (measured with ESS and Likert fatigue scores), which also reflected the effect on daily life (measured with FOSQ) in comparison with the controls. There are no objective surrogate markers for fatigue, and therefore, we used questionnaires and self-reporting, which has limitations. A history of a brain infection might lead to increased risk of over-reporting symptoms, a phenomenon described after traumatic brain injury [33]. Surprisingly, there was a correlation between older age and a high FOSQ score, which illustrates the difficulties with self-reporting of symptoms in the absence of objective markers for disease. Psychology research implies, that despite decreasing general energy levels with higher age, other circumstances among older people, such as the selection of activities suitable for their preferences and resources and ending of social interactions that are perceived dissatisfactory, can lead to a perception of the same amount of energy in the elderly as in the younger [34]. In our previous study, where the patient and control groups were larger but the age and sex distribution similar to the current study, there were no significant differences in the functional outcome measured by FOSQ, except for in the dimension *social outcome*. The follow-up time after TBEV infection differed and was in median 5,5 years in the previous study, compared to 2 years in the present, which may have provided a longer period to heal the brain injuries caused by TBE [7].

We hypothesize that persons with a history of TBE are more prone to experience symptoms from a mild degree of OSA, probably a consequence of the brain infection with its immunopathological reactions and effect on brain cells [35, 36] The potential mechanisms for the origin of fatigue in TBE might be related to neurotransmitter dysfunctions as proposed for fatigue observed in multiple sclerosis [37]. Furthermore, the areas of the brain involved in TBE might be of importance to explain fatigue and sleep disturbances as TBE sequelae. Unfortunately, clinical data on MRI findings were not available in our patients, hence, a question for the future is whether there is an association between pathology in certain areas of the brain and sleep abnormalities in TBE as well as excessive daytime sleepiness and functional outcomes.

Interestingly, the TBE cases demonstrated lower oxygenation levels (ODI, time spent below 90% SpO_2_, and min SpO_2_) in spite of having a similar OSA severity in terms of AHI. Due to the small sample size, we have no explanation for this finding but may speculate that TBE may have influences on physiological traits, such as pharyngeal collapsibility/compensation, arousal threshold and loop gain, which have been suggested to play an important role on the pathogenesis as well as the severity of OSA [38].

The controls of our study were well matched to the patient group, regarding age, sex, BMI, waist/hip ratio, smoking and alcohol consumption habits. Concomitant diseases associated with OSA were slightly more common in the control group than in the patient group—if this was not the case, we speculate that the proportion of OSA would have been higher in the TBE compared with the control group. The control persons had no history of previous TBE, and we did not test for TBEV antibodies in the control group, consequently there might be a risk of a previous asymptomatic/ subclinical TBEV infection in the control group. Severe disease is associated with a higher frequency of sequelae [6], and it is unlikely that asymptomatic/ subclinical infections would lead to significant cerebral impairments, albeit not studied. Furthermore, there are several pitfalls with serology testing in a control group. First of all, the presence of IgG antibodies against TBEV may be a result from previous infection and/or vaccination. Recently, it has been suggested that the presence of TBEV non-structural (NS)-1 antibodies may discriminate between TBEV-infected and TBEV-vaccinated individuals [39, 40], but this data has been challenged [41], and presently, there is no standardized way of determining whether detected TBEV-specific IgG antibodies are a consequence of infection or vaccination. Secondly, the interpretation of the serology test is complicated since the result can be influenced by cross-reacting antibodies from other flaviviruses by vaccination or infection. Therefore, a travel and documented vaccination history, which we did not have access to, is necessary for interpretation.

We should acknowledge a number of limitations of the current study: Firstly, the sample size of the study population was relatively small, and a power estimation was not conducted. Since the total FOSQ scores did not differ significantly between the TBE cases and the controls in our previous study with a larger sample of 96 cases and 76 controls [7], we initially planned an explorative study to address occurrence of OSA and its association with the functional outcomes among the TBE cases. Secondly, 9 out of 42 study participants underwent PSG in their home environment, and a possible “first night effect” for the PSG studies done in hospital has not been accounted for. However, this did not seem to affect the outcomes of the study, since the total sleep time did not differ significantly from the participants who underwent PSG in the hospital. Similar to our experience, a review of randomized studies comparing home—PSG with laboratory—PSG concluded that home—PSG is equally effective for sleep evaluation [42]. Finally, the subjective sleepiness was on an ESS threshold, which may not reflect a true objective sleepiness. Other methods such as Multiple Sleep Latency Test [43], which is a time-consuming but an important tool to measure the level of daytime sleepiness would have been much valuable. This test has a particular importance to verify or rule out the narcolepsy diagnosis, which may have implications in cases with post infectious encephalitis, or as suggested by some case reports, post TBEV vaccinations [44].

## Conclusions

Despite the small sample size, our study points out some valuable new findings. TBE was associated with impaired sleep-related functional outcomes independent of age, sex, total sleep time and severity of OSA. The occurrence of OSA was not higher among the adults with a history of TBE than those without TBE. However, the cases with a TBE history and concomitant OSA had the worst functional outcomes compared with the other subgroups. Given the fact that OSA is a treatable condition, further studies are warranted to determine the effect of treatment of concomitant OSA on functional outcomes with regard to optimal rehabilitation of TBE.

## Supporting information

S1 Data (XLSX)
